# Effect of porous polycaprolactone beads on bone regeneration: preliminary *in vitro* and *in vivo* studies

**DOI:** 10.1186/2055-7124-18-18

**Published:** 2014-11-24

**Authors:** June-Ho Byun, Han A Reum Lee, Tae Ho Kim, Jin Ho Lee, Se Heang Oh

**Affiliations:** Department of Oral and Maxillofacial Surgery, Institute of Health Sciences, Gyeongsang National University School of Medicine, Jinju, 660-702 Korea; Department of Nanobiomedical Science & BK21 PLUS NBM Global Research Center for Regenerative Medicine, Dankook University, Cheonan, 330-714 Korea; Department of Advanced Materials, Hannam University, Daejeon, 305-811 Korea

**Keywords:** Bone filler, Femur, Bone defect, Polycaprolactone (PCL), Porous bead

## Abstract

**Background:**

For the effective bone regeneration with appropriate pathological/physiological properties, a variety of bone fillers have been adapted as a therapeutic treatment. However, the development of ideal bone fillers is still remained as a big challenge in clinical practice. The main aims of this study are i) fabrication of a highly porous PCL beads; and ii) the estimation of the potential use of the porous PCL beads as a bone filler through preliminary animal study.

**Results:**

The porous PCL beads with size range of 53 ~ 600 μm (425 ~ 500 μm dominantly) are fabricated by a spray/precipitation method using a double nozzle spray and PCL solution (in tetraglycol). The PCL beads show highly porous inner pore structure and the pores are interconnected with outer surface pores. For the preliminary animal study, we recognize that the porous PCL bead can induce the new bone formation from the outer surface of bone defect toward the bone marrow cavity through the bead matrix.

**Conclusions:**

From the preliminary results, we can suggest that the highly porous PCL beads may be a promising candidate as a bone filler (scaffolding matrix) for the effective bone regeneration.

## Background

Although an injury bone can be reconstructed spontaneously, large bone defects created by trauma, tumor resection, corrective osteotomy, and congenital deformity are considered as a notable challenge for orthopedic and oral/maxiallofacial surgeons [1]. For the effective bone regeneration with appropriate pathological/physiological properties, a variety of bone grafts including biological and synthetic biomaterials have been utilized as a therapeutic treatment. The biological grafts (autograft and allograft) are commonly used as a first line therapy for large-sized bone defect, but insufficient donor materials, inevitable donor site morbidity, risk of infection (autograft); and risk of immune response/disease transmission (allograft) remain as significant limitations in the clinical practice [2–4]. To solve the limitations, ceramic-based materials with similar mineral constituent of bone, such as hydroxyapatite (HA) and tri-calcium phosphate (TCP) have been utilized for the effective bone regeneration due to their biocompatibility, non-immunogenecity, osteoconductivity, bonding affinity with host bone, etc. [4–8]. However, their low reliability (i.e., weak mechanical strengths and high fragile failure rate) in wet environment which leads to difficulty for load-bearing applications and long-term degradation rate which can prohibit new bone growth into the defect site are considered as a limitation for clinical applications [5, 9, 10]. Recently, US Food and Drug Administration (FDA) approved biodegradable polymers [e.g., poly(glycolic acid) (PGA), poly(lactic acid) (PLA) and poly(lactic acid-co-glycolic acid) (PLGA), poly(*ϵ*-caprolactone) (PCL), polydioxanone (PDO)] with biocompatibility, predictable degradation rate and controllable mechanical properties are gained increasing interest as alternative matrices for bone regeneration [11]. Among them, the PCL is considered as a more promising matrix for bone regeneration compared to the other biodegradable polymers because of its no acidic by-products formation during degradation, flexibility (vs. PGA, PLA, PLGA); and relatively long-term structural stability which can provide a frame work during bone regeneration (vs. PGA, PLGA, PDO). Low et al. [12] demonstrated that the PCL matrix can allow biomimetic environment for the initial blood coagulation, cell infiltration, new blood vessel formation, and effective long-term osteogenesis. Moreover, Schantz et al. [13] reported that the PCL matrix is well tolerated *in vivo* and integrated with the host bone, suggesting that the PCL matrix may be a suitable graft for bone regeneration. Nevertheless the encouraging results, the use of PCL matrix as a bone filler is still limited, probably due to the concern about long-term remaining at applied site of dense PCL matrix which may prevent new bone formation. However, we expected that the highly porous PCL matrix may allow an appropriate environment for initial bone growth (by structural stability), accelerated degradation (by large surface area), sustained delivery of bioactive molecules (by high porosity), and thus become a good candidate as a bone filler.

Therefore, the main aims of this study are i) fabrication of a highly porous PCL bead; and ii) the estimation of the potential use of the porous PCL bead as a bone filler through preliminary animal study. To achieve this goal, porous PCL beads are fabricated by a spray/precipitation method using a double nozzle spray and PCL solution (in tetraglycol). The tetraglycol which is frequently utilized in parenteral delivery [14–16] is used as a nontoxic solvent for PCL. The preliminary animal study (femur defect rat model) to estimate the bone regeneration behavior by the porous PCL bead is also investigated.

## Method

### Materials

PCL (Mw 80,000 Da) and tetraglycol (glycofurol) as a nontoxic solvent for PCL were purchased from Sigma-Aldrich (USA). All other chemicals were analytical grade and were used as received. Ultrapure grade water (>18 mΩ) was purified using a Milli-Q purification system (Millipore Co., USA). For animal study, the porous PCL beads were sterilized by ethylene oxide (EO).

### Preparation of porous PCL beads

**Figure 1:**
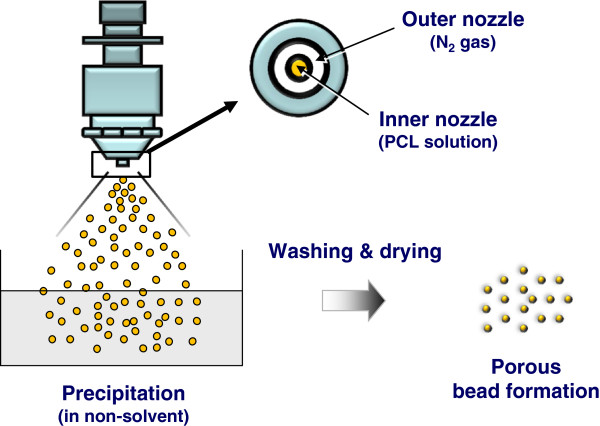
**Schematic diagram showing the porous PCL beads fabrication procedure.**

Porous PCL beads were simply prepared by spray/precipitation method using double nozzle spray. PCL pellets were dissolved in tetraglycol at 90°C (15 wt%), and the PCL solution was immediately transferred in a 10 mL syringe. The warm solution was sprayed through a double nozzle spray with N_2_ purging of 2.5 L/min (outer nozzle) into 50% ethanol solution (coagulation solution) to induce the solidification (precipitation) of PCL solution (Figure 1). Feeding rate of the solution was fixed to 60 mL/h (inner nozzle, using syringe pump). The syringe and double nozzle spray were heated (90°C) using a heating system equipped with heating tape (PID temperature controller, Model, TC130P; heating tape, Model, HT2510; Misung Scientific, Korea) to prevent precipitation of PCL during the process. The distance of tip-to-coagulation solution was 20 cm. The precipitated PCL beads were maintained at coagulation solution for 6 hrs, then the PCL beads were washed out in excess water for 24 hrs to remove residual tetraglycol and ethanol. The PCL beads were obtained by centrifugation and dried in a vacuum oven overnight, and the beads were separated in different size ranges (53 ~ 100, 100 ~ 200, 200 ~ 300, 300 ~ 425, 425 ~ 500, 500 ~ 600 μm) using standard testing sieves (Chunggye Industrial Co., Korea).

### Characterization of porous PCL beads

#### Morphology observation and porosity measurement

The morphology of prepared porous PCL beads was observed by a field emission scanning electron microscope (FE-SEM; Model S-4300, Hitachi, Japan). The cross-sectional specimen was prepared by cutting them using a blade after being frozen in liquid nitrogen. The porosity of the PCL beads was estimated using mercury porosimetry (Poresizer 9320; Micromeritics, USA). To determine the porosity, it was assumed that the surface tension of mercury is 480 dyne/cm [17].

#### Preliminary animal study

**Figure 2:**
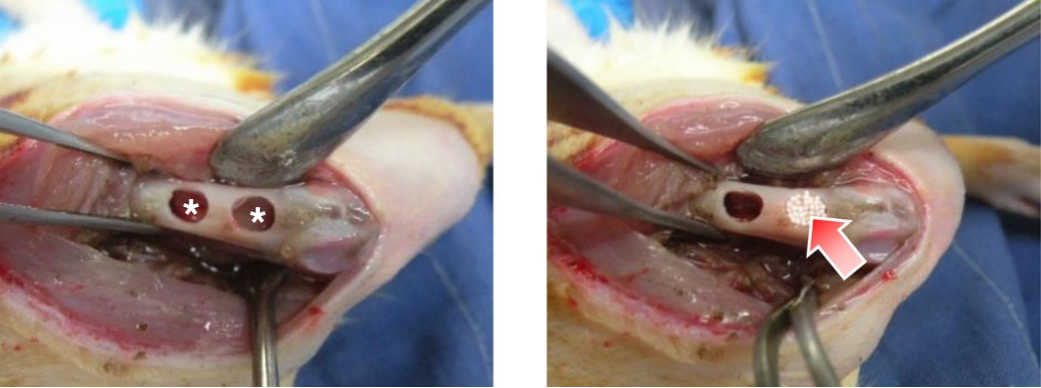
Photographs showing the implantation of porous PCL beads in a femoral defect of rat (*, bone defect; arrow, porous PCL beads).

Sprague–Dawley (SD) rats (~250 g) were selected as an animal model to estimate the bone reconstruction potential by the use of porous PCL beads. The animal study was permitted from the Animal Care Committee of the Hannam University in Korea, and all surgical procedures were performed according to the guidelines. The rats were completely anesthesized with intramuscular injection of tiletamine/zolazepam (10 mg/kg; Zoletil 50®, Virbac Laboratories, France) and 2% xylazine hydrochloride (2 mg/kg; Rumpun®, Byely, Korea), and placed in the prone position. The left leg was shaved and the skin surface discontaminated with 7% tincture of iodine. The front skin of the mid-femur in rats was incised straight and longitudinally at 30 mm in length. After splitting the muscle, the periosteum was stripped to expose the femoral bone surface. Two drill-holes (each hole, ~4 mm in diameter) were created through the femoral cortex using a small tungsten carbide dental bur with a diameter of 0.8 mm in the anterior portion of the diaphysis of one femur (10 mm position from the knee joint) (Figure 2). The bone defect was prepared with a very gentle surgical technique and continuous internal cooling with physiological saline solution. Then the porous PCL beads were implanted into the defect, and the wound was closed in two layers using 4–0 vicryl and 4–0 nylon sutures. The blank (w/o any treatment) was also studied as a control group. During surgery and 24 hrs later, all animals received subcutaneous injections of antibiotics (sulfadoxin and trimethoprin, 5:1, 15 mg/kg) to minimize the risk of infections. At 4 weeks after implantation, the rats were sacrificed by an overdose of CO_2_ gas. For histological observation, the bone defects with surrounding femoral bone were harvested, and the specimens were fixed with 4% formaldehyde and decalcified in 10% formic acid. After dehydration procedure of the fixed specimens in a graded series of alcohol, the specimens were embedded in paraffin and cut into 5 μm transverse sections in the defects. The sections were stained with Hematoxyline & eosin (H&E) and Masson’s trichrome (MT) for observation by light microscopy (Model BX50F4, Olympus). For radiographic evaluation, the tissue specimens harvested from the femur of rat were frozen, and placed on an automatic axially-moving turntable to scan using a micro-computed tomography (μ-CT) system (Skyscan 1176, Skyscan N.V., Belgium)).

## Results and discussion

### Preparation and characterization of porous PCL beads

**Figure 3:**
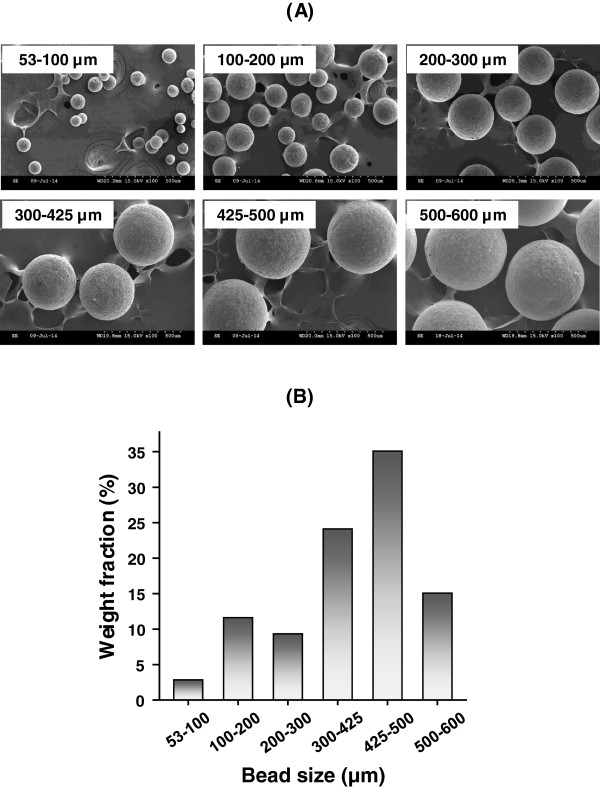
**(A) SEM Photographs of the prepared porous PCL beads with different size range (x100) and (B) their distribution expressed by weight fraction.**

**Figure 4:**
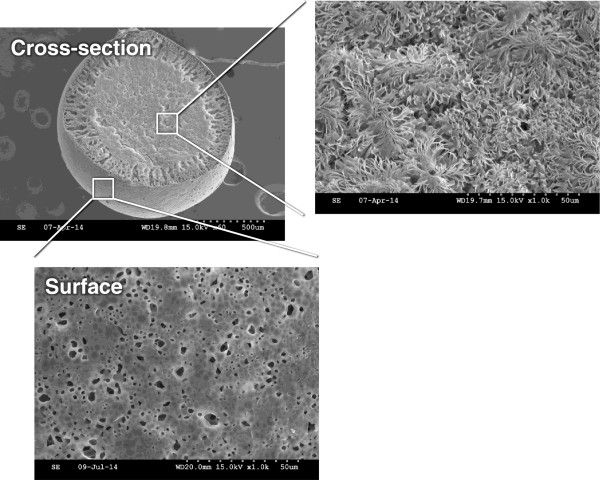
**SEM Photographs of the surface and cross-sectional view of the PCL beads with size range of 425 ~ 500 μm.**

Porous PCL beads were prepared to estimate its feasibility as an bone filler for the effective bone regeneration. The PCL is the most commonly utilized biodegradable polyesters with biocompatibility and predictable degradation rate for medical applications [18]. Their degradation is understood as a metabolism via tricarboxylic acid (TCA) cycle [19]. The porous PCL beads were fabricated by a simple spray/precipitation method using double nozzle spray and PCL solution. The gross appearance of prepared porous PCL beads with different size ranges and their surface/cross-sectional morphologies observed by SEM were shown in Figures 3 and 4.

It was observed that the size range of the prepared porous PCL beads in our procedure [purging rate of N_2_, 2.5 L/min (outer nozzle) and feeding rate of PCL solution, 60 mL/h (inner nozzle)] is 53 ~ 600 μm and the porous PCL beads with size range of 425 ~ 500 μm is more dominant than other size ranges. Their size distribution also can be controlled by the purging rate of N_2_ and/or feeding rate of polymer solution [higher N_2_ purging rate, smaller size dominantly (lower volume ratio of polymer solution to N_2_ gas); higher PCL solution feeding rate, larger size dominantly (higher volume ratio of polymer solution to N_2_ gas); not shown data), as expected. The porous PCL beads exhibited highly porous inner pore structures and the pores are interconnected with surface pores. The formation of porous structure can be understood by phase separation between polymer (PCL) solution and nonsolvent [solvent (tetraglycol)-nonsolvent (50% ethanol) exchange] during the precipitation of the PCL solution in coagulation bath [20]. From the morphology results, we could expect that the highly porous structure can provide large surface area of PCL matrix and thus may accelerate the degradation rate which can allow appropriate space for new bone formation, moreover the porous beads may be a reservoir of bioactive molecules for bone regeneration (e.g., drugs, growth factors, cytokines, etc.). Therefore, we believed that the porous PCL beads may be a promising matrix for effective bone regeneration. The porosity of the porous PCL beads measured using mercury porosimetry were over 80%, regardless of bead size. The porous PCL beads with size range of 425 ~ 500 μm were selected for the preliminary animal study using rat model [21].

### Preliminary animal study

**Figure 5:**
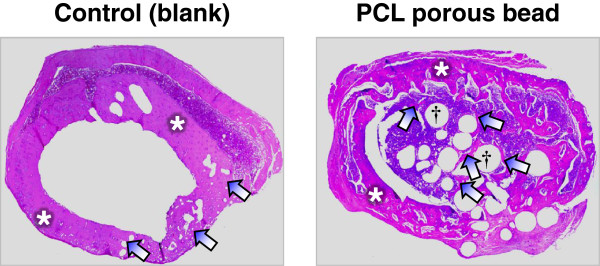
Histological sections of bone defect and surrounding femoral tissue showing the bone regeneration behavior of the control (blank) and porous PCL beads (*, host bone; arrow, new bone; †, PCL beads; H&E staining, x40).

**Figure 6:**
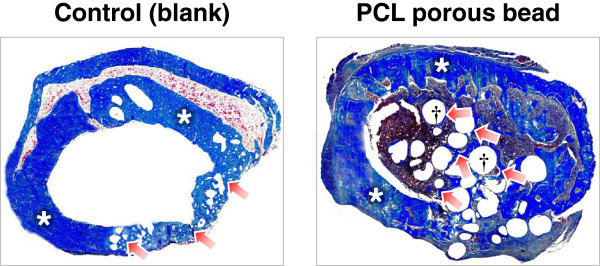
Histological sections of bone defect and surrounding femoral tissue showing the bone regeneration behavior of the control (blank) and porous PCL beads (*, host bone; arrow, new bone; †, PCL beads; Masson’s trichrome staining, x40).

**Figure 7:**
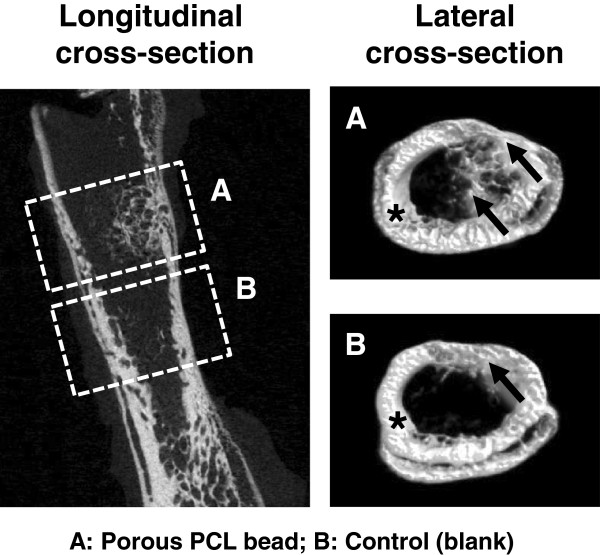
μ-CT images of femoral bone defect showing the mineralized bone regeneration behavior of (A) porous PCL beads and (B) control (blank) [*, host bone; arrow, regenerated (mineralized) bone].

A SD rat model was used to estimate the bone regeneration behavior of the porous PCL beads. The rat femoral bone defect was chosen as the orthotopic model for this experiment. Bone defects of ~4 mm diameter were created on a femur using a small tungsten carbide dental bur, and the porous PCL beads were filled into the defect. The blank (w/o any treatment) was also studied as a control group. At implantation, the porous PCL beads were easily applied and formed well-packed structure in the defect site without floating by bleeding. During the breeding, all animals remained in sound health and did not show any wound complications. Figures 5 and 6 show the histological sections (after H&E and MT staining) to compare bone reconstruction behavior in the bone defect filled with and without the porous PCL beads at 4 weeks after surgery. In the PCL bead group, the new bone was regenerated from the outer surface of bone defect toward the bone marrow cavity through the porous PCL beads. However, in the control (blank) group, the new bone was only reconstructed at outer surface of bone defect and the new bone formation in the marrow cavity was not detected. This indicates that the porous PCL bead itself can provide an appropriate environment for ingrowth of a variety of cells related with bone formation, and thus allow bone regeneration in the PCL bead matrix. The regenerated bone was grown along the porous matrix consisted of the PCL beads, suggesting that the beads are stably kept their porous structure without degradation during the bone regeneration and act as a scaffolding matrix which can allow the adhesion of bone-related cells and thus improve osteogenesis [22–24]. At 4 weeks after implantation, the mineralized bone regeneration was also studied using μ-CT. The growth of mineralized bone tissues begun at the outer surface of all bone defects (Figure 7), and the mineralized bone tissue was infiltrated through the PCL bead matrix. This observation was consistent with the result of the histological results, showing that the porous PCL beads can effectively induce the bone regeneration. On the basis of our findings, we can suggest that the highly porous PCL beads fabricated by simple spray/precipitation method may be a candidate as a bone filler for the effective bone regeneration.

## Conclusions

We prepared porous PCL beads by a spray/precipitation method using a double nozzle spray and PCL solution (in tetraglycol). It was observed that the size range of prepared porous PCL beads (purging rate of N_2_, 2.5 L/min and feeding rate of PCL solution, 60 mL/hr) is 53 ~ 600 μm (dominant size range, 425 ~ 500 μm) and their size distribution can be controlled by the purging rate of N_2_ and/or feeding rate of polymer solution. The porous PCL beads showed highly porous inner pore structure and the pores are interconnected with outer surface pores. For the preliminary animal study, we recognized that the porous PCL bead can induce the new bone formation from the outer surface of bone defect toward the bone marrow cavity through the bead matrix. From the preliminary results, we could suggest that the highly porous PCL beads may be a promising candidate as a matrix for the bone regeneration. The long-term studies (i.e., *in vivo* degradation rate of the porous PCL beads and bone regeneration/maturation behaviors through the matrix) using a large animal (porcine) to confirm the potential use of the porous PCL beads as a clinically applicable bone filler are in progress.

## Availability of supporting data

The data sets supporting the results of this article are included within the article.
